# Sex Differences in Healthspan Predict Lifespan in the 3xTg-AD Mouse Model of Alzheimer’s Disease

**DOI:** 10.3389/fnagi.2018.00172

**Published:** 2018-06-12

**Authors:** Alice E. Kane, Sooyoun Shin, Aimee A. Wong, Emre Fertan, Natalia S. Faustova, Susan E. Howlett, Richard E. Brown

**Affiliations:** ^1^Department of Pharmacology, Dalhousie University, Halifax, NS, Canada; ^2^Department of Psychology and Neuroscience, Dalhousie University, Halifax, NS, Canada; ^3^Division of Geriatric Medicine, Department of Medicine, Dalhousie University, Halifax, NS, Canada

**Keywords:** frailty, sex differences, deficit index, frailty index, deficit accumulation

## Abstract

Mouse models of Alzheimer’s disease (AD) exhibit marked differences in life expectancy depending on their genotype and sex. The assessment of frailty could provide a measure of healthspan to facilitate comparisons between different AD models. We used a validated mouse frailty index (FI) assessment tool to explore genotype and sex differences in lifespan and healthspan of 3xTg-AD mice and their B6129F2 wild-type (WT) controls. This tool is based on an approach commonly used in people and quantifies frailty by counting the accumulation of age-related health deficits. The number of deficits in an individual divided by the total number measured yields an FI score theoretically between 0 and 1, with higher scores denoting more frailty. Male 3xTg-AD mice aged 300–600 days had higher FI scores (Mean FI = 0.21 ± 0.03) than either male WT (Mean FI = 0.15 ± 0.01) or female 3xTg-AD mice (Mean FI = 0.10 ± 0.01), and the elevated frailty scores were accompanied by parallel increases in mortality. Frailty increased exponentially with age, and higher rates of deficit accumulation elevated mortality risk in all groups of mice. When mice were stratified by FI score, frailty predicted mortality, at least in females. Therefore, the mouse clinical FI provides a valuable tool for evaluating healthspan in mouse models of AD with different lifespans.

## Introduction

Alzheimer’s disease (AD) is the most common type of dementia and its prevalence is increasing as the population ages (Mielke et al., 2014; Scheltens et al., 2016). Since the early 2000s, there has been considerable interest in the development of mouse models to study the mechanisms underlying the development and progression of AD, and to investigate potential therapies (Chin, 2011; Webster et al., 2014; Esquerda-Canals et al., 2017). There are now over 190 purported transgenic mouse models of AD^1^. One of the most widely used strains is the 3xTg-AD mouse model (Oddo et al., 2003; Sy et al., 2011) which has three gene mutations (APP-Swedish, PS1M146V and tauP301L), resulting in a phenotype with amyloid beta plaques and tau pathology in the brain as well as deficits in cognitive function (Oddo et al., 2003; Billings et al., 2005; Stevens and Brown, 2015; Stover et al., 2015). Although there has been some criticism of AD mouse models in general (Onos et al., 2016) and the 3xTg-AD model in particular (Hargis and Blalock, 2017; Sasaguri et al., 2017), the 3xTg-AD mouse has been considered one of the most important models of AD as it develops both plaques and tangles (Webster et al., 2014; Esquerda-Canals et al., 2017).

There are considerable genotype and sex differences in life expectancy among inbred mouse strains and among the different mouse models of AD (Rae and Brown, 2015; Brown et al., 2018). Although many AD mouse models have shorter lifespans than their wild-type (WT) controls, this is not observed in all models (Rae and Brown, 2015). There are also sex differences in the lifespan of some AD mouse models, including 3xTg-AD mice, in which males have a shorter lifespan than females (Westmark et al., 2010; Rae and Brown, 2015). In some models, however, males have longer lifespans than females (Pugh et al., 2007), or there is no sex difference (Rae and Brown, 2015; Brown et al., 2018). This makes it difficult to compare the lifespan and interventional data across different AD mouse models and between the sexes, as well as the translation of data from animal studies to the clinic. Therefore, it would be beneficial to have a standard measure of biological age or healthspan that is distinct from chronological age that could be used across mouse models (Rae and Brown, 2015). The consideration of healthspan, not just lifespan, in all longevity and aging studies is essential in the field of aging research (Howlett and Rockwood, 2014; Richardson et al., 2015; Huffman et al., 2016).

In humans, the frailty index (FI) is a commonly used measure of overall health status and resilience as it is considered a measure of a person’s biological age, as distinct from their chronological age (Rockwood and Mitnitski, 2007; Clegg et al., 2013). Tools to assess frailty in mice have been developed and validated (Parks et al., 2012; Liu et al., 2014; Whitehead et al., 2014; Feridooni et al., 2015; Antoch et al., 2017; Gomez-Cabrera et al., 2017; Rockwood et al., 2017). The mouse clinical frailty index (MCFI) was the first of these tools, and has been used in a variety of studies (Whitehead et al., 2014; Kane et al., 2016a,b; Huizer-Pajkos et al., 2016; Moghtadaei et al., 2016; Jansen et al., 2017). The MCFI is based on the FI approach that is commonly used to quantify frailty in humans (Kane and Howlett, 2018). The FI counts the number of health-related deficits a person or mouse has accumulated and the total is divided by the number of deficits assessed, resulting in a FI score between 0 and 1. A higher FI score indicates a greater degree of frailty and is considered a measure of healthspan in humans (Searle et al., 2008). The MCFI is a non-invasive assessment of 31 health-related deficits across a range of domains and is considered a measure of healthspan in mice (Whitehead et al., 2014; Seldeen et al., 2015). High scores in the MCFI have been associated with increased mortality for inbred C57BL/6 mice (Rockwood et al., 2017), but the MCFI has not been used to examine the healthspan of genetically modified mice or transgenic mouse models of AD.

In humans, there are important sex differences observed in both AD and frailty. AD is more common in women (Mielke et al., 2014; Mazure and Swendsen, 2016), and men and women often have different presentations of the disease (Sinforiani et al., 2010; Mazure and Swendsen, 2016). Despite this, many studies show that women have a greater life expectancy with AD than men (Sinforiani et al., 2010; Mielke et al., 2014; Mazure and Swendsen, 2016). In terms of frailty, women have higher FI scores than men at all ages, but, paradoxically, have lower mortality risk (Mitnitski et al., 2002; Puts et al., 2005; Yang and Lee, 2010; Gordon et al., 2017). The FI is also related to cognitive decline in Alzheimer’s patients as increases in the FI are correlated with increased cognitive decline and a higher probability of converting from mild cognitive impairment (MCI) to AD (Kelaiditi et al., 2016; Trebbastoni et al., 2017). Increasing age, male sex and higher FI scores were associated with a higher probability of converting from MCI to AD in patients (Trebbastoni et al., 2017).

In mice, along with genotype and sex differences in mortality in different AD models, there also appear to be sex differences in frailty. Older C57BL/6 female mice have higher FI scores than age-matched males (Whitehead et al., 2014; Antoch et al., 2017), although this has not been shown in all studies (Parks et al., 2012; Kane et al., 2016a). The association of age-related changes in FI scores with mortality in mouse models of AD has not yet been explored, but is of great interest (see Seldeen et al., 2015; Kelaiditi et al., 2016). Thus, the aim of this study was to use the MCFI to assess genotype and sex differences in age-related changes in frailty in male and female 3xTg-AD mice and their WT controls to determine the relationship between healthspan and lifespan.

## Materials and Methods

### Animals

The 3xTg-AD mice (*n* = 35 male, *n* = 62 female) and B6129SF2 WT mice (*n* = 84 male, *n* = 91 female) were bred at Dalhousie University from parents originally purchased from the Jackson Laboratory (Bar Harbor, ME, USA, 3xTg-AD, JAX#004807; B6129SF1/J, JAX #101043). Mice were weaned at 21 days of age, and housed in groups of 2–4 same-sex littermates in plastic cages (18.75 × 28 × 12.5 cm) with a PVC tube (4 cm diameter × 7 cm length) for enrichment, woodchips for bedding and metal wire covers. Animals were maintained on a reversed 12:12 light–dark cycle with lights off at 9:30 am, and *ad libitum* access to food (Purina #5001) and water. Frailty testing was completed during the dark phase of the light-dark cycle. All experimental procedures were conducted in accordance with the guidelines published by the Canadian Council on Animal Care and were approved by the Dalhousie University Committee on Laboratory Animals (#15-099).

Four groups of mice were compared in these experiments: female WT, male WT, female 3xTg-AD and male 3xTg-AD. Mice were followed over their lifespan for survival. Date of death was determined as the day a mouse was found dead or determined to be moribund by a veterinarian. Some mice were used for separate experiments and these were included in the longevity analysis as censored data. For some mice the date of death was unknown, and these mice are included in frailty analyses but excluded from survival analyses (female WT *n* = 7, male WT *n* = 8, female 3xTg-AD *n* = 8, male 3xTg-AD *n* = 2).

### Mouse Clinical Frailty Index Assessment

Mice were assessed using the MCFI (Whitehead et al., 2014; Feridooni et al., 2015), which includes 31 “clinically” assessed non-invasive items. For 29 of these items, mice were given a score 0 if not present, 0.5 if there was a mild deficit, and 1 for a severe deficit. The final two items were weight and body surface temperature, which were scored based on the number of standard deviations from a reference mean in young adult mice as previously described (Whitehead et al., 2014). Each mouse was assessed with the MCFI at one time point and days to death post FI measurement were determined. For some analyses FI scores were stratified as either high (≥0.21) or low (<0.21), using a cut-off point that is commonly used clinically (Rockwood et al., 2011; Blodgett et al., 2015), and has been used previously in rodents (Yorke et al., 2017). To compare the rates of deficit accumulation in different sex and genotype groups in the present study, the natural log of the FI was plotted against age. The slope of this line provides an estimate of the rate of deficit accumulation, as shown in previous studies (Whitehead et al., 2014; Yorke et al., 2017).

### Statistics

Data are shown as mean ± SEM unless otherwise indicated. Kaplan Meier (KM) curves were plotted to show survival probabilities over time in genotype and sex groups. Male and female TG and WT mice were also divided into low and high FI score groups (<0.21 and ≥0.21), and survival for each genotype and sex group was plotted on KM curves. Log rank analyses were used to determine if there were differences between the KM curves. The correlation between FI score, or the natural log of FI score, and age was determined for each group with Pearson correlation statistics. Mean FI scores, age at FI score and lifespans were compared between groups using a 2-way analysis of variance (ANOVA) for genotype and sex. An ANCOVA of sex/genotype group with age as a covariate was used to compare the slopes of ln FI vs. age. Mean FI scores were determined for each genotype and sex group in three different age groups: 0–300 days, 300–600 days and more than 600 days old. A 3-way ANOVA was used to determine the effect of genotype, sex and age on FI scores. Two-way ANOVAs were used to determine the effect of genotype/sex, age and the interaction of these variables on FI scores. Bonferonni *post hoc* analyses were used for all ANOVAs. Data analyses were completed using the SPSS statistics program (Version 21.0, SPSS Inc., Chicago, IL, USA) and SigmaPlot (Version 11.0, Systat Software, Germany). *P* values less than 0.05 are considered significant.

## Results

### Survival of Male and Female 3xTg-AD and WT Mice

**Figure 1:**
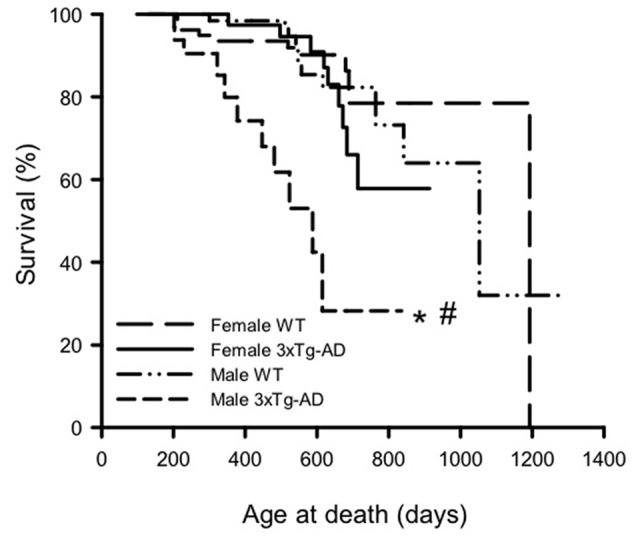
Kaplan-Meier survival curves for male and female 3xTg-AD and wild-type (WT) mice. Log rank analyses were used to compare survival curves (*p* < 0.001). Pairwise analyses show that male 3xTG-AD mice (*n* = 33) were significantly shorter lived than both male WT mice (*n* = 76), and female 3xTG-AD mice (*n* = 54; *p* < 0.001). By contrast, there was no significant difference in survival between the female WT (*n* = 84) and 3xTg-AD mice. *p < 0.05 compared to corresponding female group, ^#^*p* < 0.05 compared to corresponding WT group.

KM survival curves were plotted to determine differences in survival probability for male and female 3xTg-AD and WT mice (Figure 1). Log rank analyses show an overall difference in survival curves over the four groups ($\chi_{\left(3\right)}^{2}$ = 33.60, *p* < 0.001). Pairwise comparisons show that male 3xTg-AD mice have lower survival probability than male WTs ($\chi_{\left(1\right)}^{2}$ = 24.01, *p* < 0.001), but there was no difference in survival probability between female 3xTg-AD mice and WT mice. In terms of sex differences, there was no significant difference between the curves for WT male and female mice, but male 3xTg-AD mice had shorter lifespan than female 3xTg-AD mice ($\chi_{\left(1\right)}^{2}$ = 14.77, *p* < 0.001).

Mean (±SD) and maximum lifespans are summarized for each group in Table 1. Analysis of mean lifespan for each group with a 2-way ANOVA for sex and genotype showed a significant effect of genotype (*F*_(1,243)_ = 16.03, *p* < 0.001), sex (*F*_(1,243)_ = 3.79, *p* < 0.05) and their interaction (*F*_(1,243)_ = 8.44, *p* < 0.01). *Post hoc* analyses showed the same trend as the KM curves, with male 3xTg-AD mice having a significantly lower mean lifespan than male WT, and female 3xTg-AD mice (*p* < 0.05; Table 1). Maximum lifespan was lowest for male 3xTg-AD mice at 836 days, with WT mice of both sexes having similar maximum lifespans (1281 days for males and 1193 for females), and female 3xTg-AD mice having a maximum lifespan of 914 days (Table 1). Rates of censored data for each group ranged from 66.7% to 86.9%, with the male 3xTg-AD mice having the lowest rate of censoring, and highest rate of death (33.3%). Known causes of death in mice used in this study are shown in Supplementary Table S1.

**Table 1:** Summary data for lifespan and healthspan measures for each mouse group.

	Male WT	Male 3xTg-AD	Female WT	Female 3xTg-AD
**Lifespan data**				
N (survival)	76	33	84	54
Mean lifespan, days (±SD)	571.3 ± 202.1	386.9 ± 161.2^a^	548.1 ± 206.0	517.1 ± 197.1^c^
Lifespan Range (days)	280–1281	129–836	132–1193	98–914
Number (%) of mice, died	11 (14.5)	11 (33.3)	11 (13.1)	9 (16.7)
Number (%) of mice, censored	65 (85.5)	22 (66.7)	73 (86.9)	45 (83.3)
**Healthspan data**				
N (frailty)	84	35	91	62
Mean age at FI, days (±SD)	453.4 ± 212.3	286.1 ± 126.1^a^	485.3 ± 213.9	443.7 ± 196.6^c^
Mean FI (±SD)	0.14 ± 0.08	0.11 ± 0.08	0.12 ± 0.08^b^	0.11 ± 0.10
FI Range	0.01–0.50	0.02–0.40	0.0–0.39	0.0–0.48

*^a^p < 0.05 male TG compared to male WT, ^b^p < 0.05 female WT compared to male WT, ^c^p < 0.05 female TG compared to male TG*.

### Frailty Scores Over the Lifespan for Male and Female 3xTg-AD and WT Mice

The MCFI was scored in 3xTg-AD and WT male and female mice. The data that were used to construct the MCFI, grouped by systems, are presented in Supplementary Table S2. The mean FI scores for WT mice (Table 1) were comparable to those obtained in previous rodent studies (Whitehead et al., 2014; Rockwood et al., 2017; Yorke et al., 2017). Frailty research in humans has shown that there is an upper limit to FI scores that is below the theoretical maximum score of 1 (Rockwood et al., 2011, 2017; Whitehead et al., 2014; Yorke et al., 2017). The maximum FI score in the current study was 0.50 (Table 1), which is similar to the submaximal FI limit near 0.70 observed in both human and rodent FI studies (Rockwood et al., 2011, 2017; Whitehead et al., 2014; Yorke et al., 2017). FI scores were completed across the lifespan for all groups, however the mean age at FI scoring for male 3xTg-AD mice was significantly lower than the other groups, due to their significantly shorter lifespan (Table 1).

**Figure 2:**
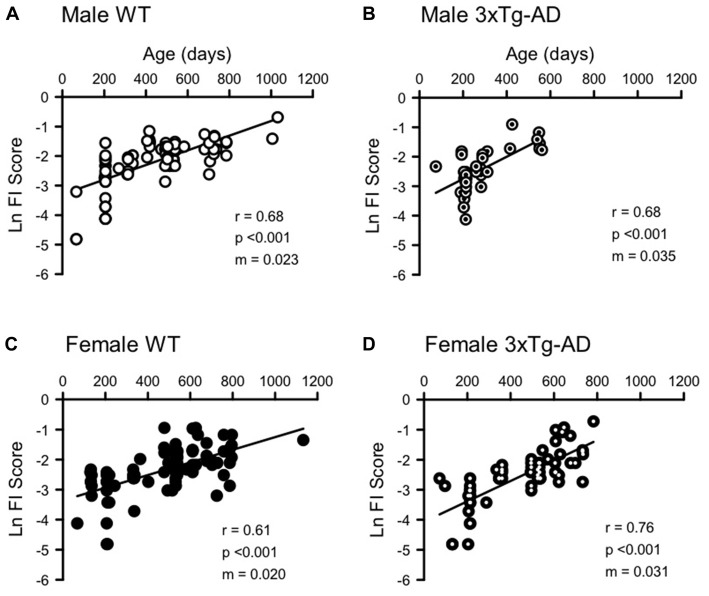
Natural log frailty index (FI) scores increase with age in male and female 3xTg-AD and WT mice. Ln FI Score was correlated with age for **(A)** WT males (*n* = 84, *r* = 0.681, *p* < 0.0001), **(B)** 3xTg-AD males (*n* = 35, *r* = 0.677, *p* < 0.0001), **(C)** WT females (*n* = 91, *r* = 0.608, *p* < 0.0001) and **(D)** 3xTg-AD females (*n* = 62, *r* = 0.755, *p* < 0.0001). The slope of the linear regression line (m, shown on each graph) is equivalent to the rate of deficit accumulation and is 0.023, 0.035, 0.020 and 0.031 for male WT, male 3xTg-AD, female WT and female 3xTg-AD respectively. A larger slope indicates a faster accumulation of deficits.

**Figure 3:**
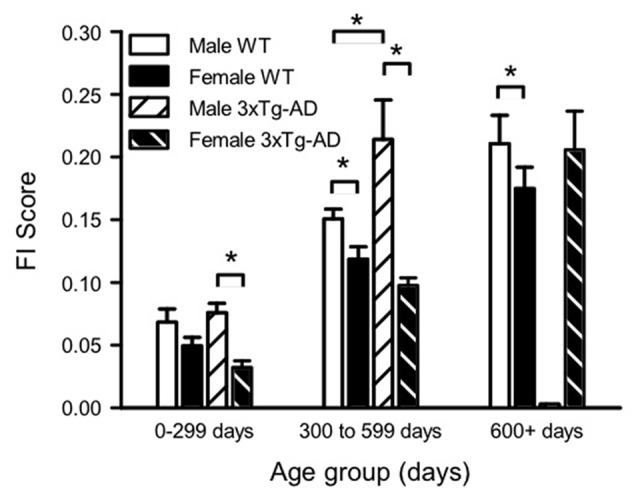
Sex and genotype differences in mean FI scores over the lifespan. Mean FI scores were plotted for each sex and genotype group at three different ages (0–300 days, 300–600 days and 600+ days). Analysis was completed with 3-way and 2-way analyses of variances (ANOVAs) with Bonferroni *post hoc* testing. Three-way ANOVA showed a significant effect of age and sex, and 2-way ANOVA showed a significant effect of genotype for males only. Two-way ANOVAs of age group and either sex or genotype showed that for all groups, mean FI scores increased with increasing age (statistics not shown on graph). *p < 0.05 between sexes. 0–300 days Male WT *n* = 23, Male 3xTg-AD *n* = 26, Female WT *n* = 22, Female 3xTg-AD *n* = 19; 300–600 days Male WT *n* = 47, Male 3xTg-AD *n* = 9, Female WT *n* = 41, Female 3xTg-AD *n* = 25; 900 + days Male WT *n* = 17, Male 3xTg-AD *n* = 0, Female WT *n* = 29, Female 3xTg-AD *n* = 17.

FI scores increased with age in all groups (Figures 2, 3, Supplementary Figure S1). Supplementary Figure S1 shows a significant exponential association between increasing age and increasing FI scores in each group, as has been shown in previous studies (Whitehead et al., 2014; Rockwood et al., 2017). To examine the rate of deficit accumulation, Figure 2 shows the natural log FI score plotted against mouse age. The natural log allows the exponential data to be plotted on a linear scale and facilitates comparison with rates of deficit accumulation in other studies. The linear correlations between age and Ln FI scores were also significant for each group (Figure 2). The slope of the linear regression line for each curve (m) is equal to the rate of deficit accumulation. An ANCOVA showed a significant difference between the slopes of each regression line, indicating different rates of deficit accumulation among genotype and sex groups (Interaction term *F* = 3.163, df = 3,260, *p* < 0.05). *Post hoc* pairwise comparisons showed that male 3xTg-AD mice have a significantly higher rate of deficit accumulation (0.035) than female 3xTg-AD (0.031) and female WT mice (0.020; Figure 2). Male WT mice (0.023) had significantly lower rates of deficit accumulation than female 3xTg-AD mice (Figure 2).

To analyze sex and genotype differences in FI scores over the lifespan, mice were binned into three age groups, and mean FI scores were calculated for each sex/genotype group at each age (Figure 3). There were no FI assessments completed on 3xTg-AD males aged older than 600 days as the majority of these mice died before then. A 3-way ANOVA of sex, genotype and age group showed a significant effect of sex (*F*_(1,262)_ = 32.14, *p* < 0.001), age group (*F*_(1,262)_ = 6.59, *p* < 0.001) and the interaction of sex and genotype (*F*_(1,262)_ = 6.59, *p* < 0.01). Two way ANOVAs of age group and either sex or genotype showed that, for all groups, mean FI scores increased with increasing age (Figure 3). The effect of genotype was significant with a 2-way ANOVA for males (*F*_(2,118)_ = 5.19, *p* < 0.05), but not females. Male 3xTg-AD mice were more frail than WT males at 300–600 days of age. A 2-way sex and age ANOVA showed that male 3xTg-AD mice were more frail than females at all ages (*F*_(1,92)_ = 23.55, *p* < 0.001, Figure 3), while male WT mice were more frail than females after 300 days of age (*F*_(1,170)_ = 9.56, *p* < 0.01, Figure 3). Overall mean FI at all ages was lower in WT females than WT males (Figure 3).

### Correlations Between FI Score and Survival Probability for Male and Female 3xTg-AD and WT Mice

**Figure 4:**
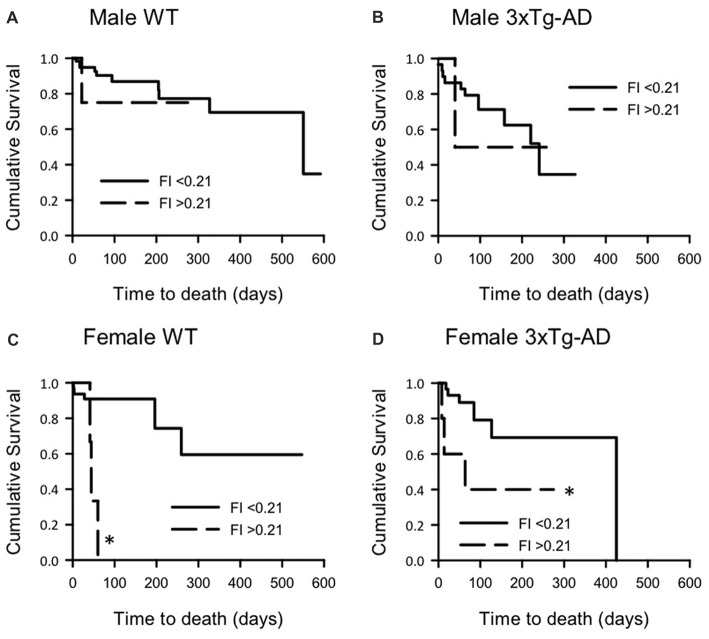
Kaplan-Meier survival curves for male and female 3xTg-AD and WT mice with low and high FI scores. Survival curves for **(A)** male WT, **(B)** male 3xTg-AD, **(C)** female WT and **(D)** female 3xTg-AD mice split into those with low (<0.21) and high (≥0.21) FI scores. Survival (days) post FI score was plotted. Log rank analysis was used to compare curves and showed that, for female mice of both groups, higher FI scores were associated with shorter survival times (*p* < 0.01). A similar trend was also seen for males but this was not statistically significant. *p < 0.05 compared to corresponding low FI group. Male WT low FI *n* = 66, high FI *n* = 10; Male 3xTg-AD low FI *n* = 30, high FI *n* = 3; Female WT low FI *n* = 74, high FI *n* = 10; Female 3xTg-AD low FI *n* = 48, high FI *n* = 6.

To determine the correlation of FI score with survival probability, each group of mice was divided into those with high and low FI scores. Days to death post FI score measurement were plotted against survival probability in KM curves (Figure 4). Overall, high FI scores were associated with higher mortality risk when compared to low FI scores. Log-rank analysis of each pair of curves showed that female WT and 3xTg-AD mice with higher FI scores had higher mortality compared to those with low FI scores (F 3xTg-AD: $\chi_{\left(1\right)}^{2}$ = 4.37, *p* < 0.05; F WT: $\chi_{\left(1\right)}^{2}$ = 8.87, *p* < 0.01; Figures 4C,D). There was no significant difference between the survival curves for either male group, although this may be due to the small numbers and high rate of censoring in the male high FI groups (High FI male 3xTg-AD: 1 died, 2 censored; high FI male WT: 1 died, 9 censored).

## Discussion

This study was the first to explore the relationship between genotype, sex and frailty in a mouse model of AD. Male 3xTg-AD mice had higher FI scores than either male WT or female 3xTg-AD mice, and these differences in frailty were accompanied by differences in mortality. Frailty increased exponentially with age in all groups, and higher rates of deficit accumulation corresponded to increased mortality risk. Finally, mortality differences in female 3xTg-AD and WT groups were revealed when groups were stratified by FI score, with higher FI scores predicting higher mortality risk.

Male 3xTg-AD mice were shorter lived than male WT mice but this genotype difference in lifespan was not seen in female mice. Additionally, both male 3xTg-AD and WT mice had higher FI scores than the corresponding female groups. Previous work in this AD mouse model has shown this sex difference in mortality risk, with male, but not female, 3xTg-AD mice showing much shorter lifespans than WT controls (Rae and Brown, 2015). Other mouse models of AD also show sex differences in mortality, with males mostly displaying shorter lifespans than females (Westmark et al., 2010; Rae and Brown, 2015). The mechanisms underlying these sex differences in mortality in mice are not understood, but may be related to differences in genetic or epigenetic factors or immune system differences (Rae and Brown, 2015). It is not clear whether sex differences in mortality in mouse models of AD indicate sex-specific phenotypes, or differences in survival in response to the same degree of pathology. The sex differences in mortality in AD mouse models appears to correspond to what is seen in humans; men with AD generally have greater mortality risk than women (Sinforiani et al., 2010; Mielke et al., 2014; Mazure and Swendsen, 2016) and have a higher FI (Trebbastoni et al., 2017). In humans, the mechanism underlying this sex difference in lifespan is not well understood but may be related to sex differences in brain development and structure, in risk factors for AD (Mielke et al., 2014), or in the pathology of AD (Mielke et al., 2014; Mazure and Swendsen, 2016).

In the current study, we showed that this sex difference in mortality was accompanied by increases in frailty scores. Male 3xTg-AD mice had higher frailty scores than male WT mice, female WT mice, and female 3xTg-AD mice across the lifespan. Thus, it appears that male 3xTg-AD mice not only had shorter lifespans, but also shorter healthspans as assessed by frailty. Higher rates of health-related deficit accumulation (frailty) also corresponded to greater mortality, with male 3xTg-AD mice showing the highest mortality rate, and female WTs the lowest. This shows, for the first time, the importance and value in measuring not just lifespan but also overall health status in studies of longevity and aging in transgenic mouse models.

In addition to sex differences in frailty in the 3xTg-AD mice, we also found that WT males had higher frailty scores than WT females in mice over 300 days of age. This higher baseline frailty in the males may explain why their mortality is generally more impacted by the AD mutations than females (Westmark et al., 2010; Rae and Brown, 2015). Factors that may contribute to higher frailty in older males include low circulating testosterone levels or reduced physical activity and sarcopenia (Saad et al., 2017), although this remains speculative. Interestingly, previous animal studies exploring sex differences in frailty using the MCFI have found either no sex differences in frailty (Parks et al., 2012; Kane et al., 2016a), or that females are more frail than males (Whitehead et al., 2014). However, these studies were all completed on C57BL/6 mice while the current study used WT mice of the B6129SF2 strain. Phenotype differences in mortality are common between mouse strains (Yuan et al., 2009, 2011; Austad and Fischer, 2016), so one might expect to find differences in FI between strains. Indeed, no sex differences in frailty were found in 18 month old DBA2/J mice (Kane et al., 2016a), and female Swiss mice were found to be more frail than males at 12–24 months of age (Antoch et al., 2017). Although in humans, females generally have higher FI scores than males, they paradoxically have a reduced risk of mortality (Gordon et al., 2017). The reason for this paradox is not known. As with humans, the association between sex, age, frailty and mortality is not well understood in mice and more studies in mouse models would improve our understanding of the interaction between these factors.

Although there were no overall differences in mortality between 3xTg-AD and WT females, when female mice were stratified by FI scores there was a correlation between MCFI scores and mortality. Female mice with higher MCFI scores, regardless of genotype, were more likely to have a reduced probability of survival. Thus, frailty is able to predict mortality risk in these mice, even though genotype was not. A similar trend was seen in male mice stratified by FI scores, although high rates of censoring (70%–90%) in the male high FI groups reduced the power to detect changes in mortality risk for these mice.

The neuropathological changes in brains of AD patients show a gradual increase over age and these changes have been classified into a series of seven stages (Braak and Braak, 1991; Braak et al., 2011). A similar series of stages has been determined for mouse models of AD (Granic et al., 2010; Hurtado et al., 2010). The progression of neuropathology in the 3xTg-AD mouse has been staged from 2 to 26 months of age in males (Mastrangelo and Bowers, 2008) and from 3 weeks to 20 months of age in both males and females (Oh et al., 2010).

Within the 3xTg-AD mice, sex differences have been found in AB plaque load and tau pathology (Billings et al., 2005; España et al., 2010; Oh et al., 2010), the cholinergic system (Perez et al., 2011); neurogenesis (Rodríguez et al., 2008); and stress hormone reactivity (Clinton et al., 2007), but in each case, females showed earlier and more severe pathology than males. Although there have been a number of studies on the role of gonadal hormones in the neuropathology of 3xTg-AD mice (Rosario et al., 2006; Carroll et al., 2007; Overk et al., 2013), it is difficult to come to any conclusions about the effects of gonadal hormones on the neuropathology of AD (Dubal et al., 2012). Pike (2017) found “compelling yet incomplete evidence that the sex-specific, age-related depletion of estrogens in women and androgens in men are significant factors in the association between age and AD” but their mechanisms of action remain unknown. Indeed, AD pathology is enhanced by gonadectomy of both male and female 3xTg-AD mice (Pike, 2017). Thus, it is possible that fluctuations in sex hormone levels contribute to sex differences in disease expression at any age.

The neuroimmune system of 3xTg-AD mice is more impaired in males than in females (Giménez-Llort et al., 2008; Arranz et al., 2011) and this might be responsible for the increased morbidity and mortality observed in male 3xTg-AD mice. There is a wide range of immune system deficits in 3xTg-AD mice and in virtually every measure, males have greater pathology than females (Kapadia et al., 2018). This sex difference in immune dysfunction increases with age, suggesting that sex-specific immunological dysfunctions in 3xTg-AD mice are age-related. Thus, it seems most likely that the increased frailty scores of 3xTg-AD mice and the sex difference observed, in which males are more impaired than females, is the result of immune system dysfunction. Additional work would be of interest, especially in view of the much higher frailty and mortality seen in males when they are investigated into old age, as shown here and previously (Rae and Brown, 2015).

Although the original 3xTg-AD publication reported a similar phenotype exists in young adult male and female mice (Oddo et al., 2003), there is some evidence that the behavioral and neuropathological phenotypes may be more severe in young adult females (Pietropaolo et al., 2008; Branca et al., 2014; Stimmell et al., 2018). In our laboratory we have studied both male and female 3xTg-AD mice throughout their lifespan. Male 3xTg-AD mice have shorter lifespans than females and both sexes have shorter lifespans than control B6129SF2/J mice (Rae and Brown, 2015).

In terms of behavioral deficits, 3xTg-AD mice showed working and memory deficits at 2–15 months of age and male 3xTg-AD mice showed more working memory and reference memory deficits than females (Stevens and Brown, 2015). The 3xTg-AD mice had impaired spatial learning and memory in the Barnes maze at 6 months of age, and male 3xTg-AD mice made more errors than the females (Stover et al., 2015). In the Morris water maze the 3xTg-AD mice had a deficit in learning as they had longer distances to reach the platform during both acquisition and reversal, which is consistent with previous research (Stover et al., 2015). Although there is at least one report of an age-dependent sex difference in 3xTg-AD mice in the Morris water maze where females have a larger deficit than males before 12 months of age, with the deficit disappearing at later ages (Clinton et al., 2007), we found no overall sex effects in learning or memory across the lifespan (Stover, 2015).

In terms of neuropathological symptoms, at 2-months of age, both male and female 3xTg-AD mice had positive staining for amyloid beta. Both male and female 3xTg-AD mice at 15-months of age had extensive positive staining for amyloid beta plaques (Fraser, 2013). Male 3xTg-AD mice at 2-months of age had almost no positive staining for tau, however females at 2-months of age had positive tau staining in both the primary motor cortex and the hippocampus CA1. By 15-months of age, both male and female mice had positive tau staining in the CA1, but females had more staining than males in the primary motor cortex (Fraser, 2013).

There are some limitations to the work presented here. There was a relatively low sample size for mice with high MCFI scores, which may have made it difficult to detect an effect of frailty on mortality in the 3xTg-AD males. This study was a cross-sectional design rather than a longitudinal study, so repeated measures of frailty scores on the same mice over the lifespan were not available. Future studies that followed male and female AD mice longitudinally with serial FI measurements would provide more information about the relationship between age, sex, frailty and AD over the lifespan of mice of different genotypes. In addition, although the MCFI does include tests of motor function, it would be interesting to include cognitive tests as part of this tool to assess healthspan. Also, as some mice in the current study were used for additional experiments, there was considerable censoring of data in the mortality analyses. Although we were still able to make statistically significant conclusions, more uncensored longevity data would increase this power even further.

Some readers may wonder why we did not employ a data reduction technique, such as a principle components analysis (PCA), to reduce the number of items in the FI and identify latent variables. Typically, this approach is resisted as a matter of principle in constructing an FI. That is because, although it is possible to do, it detracts from the central idea that there are different ways to become frail and each of them is valid for the person or mouse who becomes frail in that way. Indeed, several lines of evidence show that the dimensionality reduction that is achieved by a single variable—the FI—is preferable to a PCA that is likely to yield more variables (Song et al., 2004; Kulminski et al., 2008; Farrell et al., 2016). For the largest study to date, a PCA found that, uniquely among four leading frailty instruments tested in humans, only a 39 item FI met criteria for unidimensionality with 74% of the explained variance and 2.1% of the unexplained variance (Widagdo et al., 2016).

In summary, this study was the first to use the MCFI in a transgenic AD mouse model. The MCFI showed similar characteristics to FIs measured in other studies in rodents and humans (Whitehead et al., 2014; Rockwood et al., 2017). The FI scores in the current study increased with age in an exponential manner, had rates of deficit accumulation similar to those seen in other studies (Whitehead et al., 2014; Yorke et al., 2017), displayed a submaximal limit close to 0.70, and were related to mortality (Rockwood et al., 2017). This provides further validation of the MCFI as a valuable tool for use in longevity and aging animal studies. It also helps to explain the differential survival of male and female 3xTg-AD mice.

In conclusion, we found significant sex differences in both the lifespan and healthspan of 3xTg-AD mice. Male 3xTg-AD mice were more frail than male WT and female 3xTg-AD mice, and this was accompanied by increased mortality. Frailty increased exponentially with age in all mice, and higher rates of deficit accumulation corresponded to increased mortality. Stratification of female mice by frailty scores showed that higher MCFI scores were related to increased mortality. These results show the importance of considering the overall health status of mice, not just their chronological age. The MCFI is a valuable measure of healthspan for use in AD animal studies.

## Author Contributions

AK wrote the manuscript. SS collected the data. AW organized data and edited the manuscript. EF and NF organized data. SH and RB supervised the project and edited the manuscript.

## Conflict of Interest Statement

The authors declare that the research was conducted in the absence of any commercial or financial relationships that could be construed as a potential conflict of interest.

## Footnotes

^1^https://www.jax.org/research-and-faculty/tools/alzheimers-disease-center/alzheimers-mouse-model-repository

## Supplementary Material

FIGURE S1 Increasing FI scores are correlated with increasing age in male and female 3xTg-AD and WT mice. FI Score is exponentially correlated with age (days) for **(A)** WT males (*n* = 84, *r* = 0.474, *p* < 0.0001), **(B)** 3xTg-AD males (*n* = 35, *r* = 0.461, *p* < 0.0001), **(C)** WT females (*n* = 91, *r* = 0.377, *p* < 0.0001) and **(D)** 3xTG females (*n* = 62, *r* = 0.574, *p* < 0.0001).

TABLE S1 Causes of death or reason for euthanasia in WT and 3xTg-AD mice of both sexes. Numbers represent the number of mice in each category. Most mice were used in other experiments.

TABLE S2 Raw data used to construct a frailty index from wild-type and 3xTg-AD mice of both sexes.
