# Supplementary figures and images for: Sex Differences in Healthspan Predict Lifespan in the 3xTg-AD Mouse Model of Alzheimer’s Disease
Source: Front Aging Neurosci. 2018 Jun 12;10:172. doi: 10.3389/fnagi.2018.00172 (PMC6005856; doi:10.3389/fnagi.2018.00172)

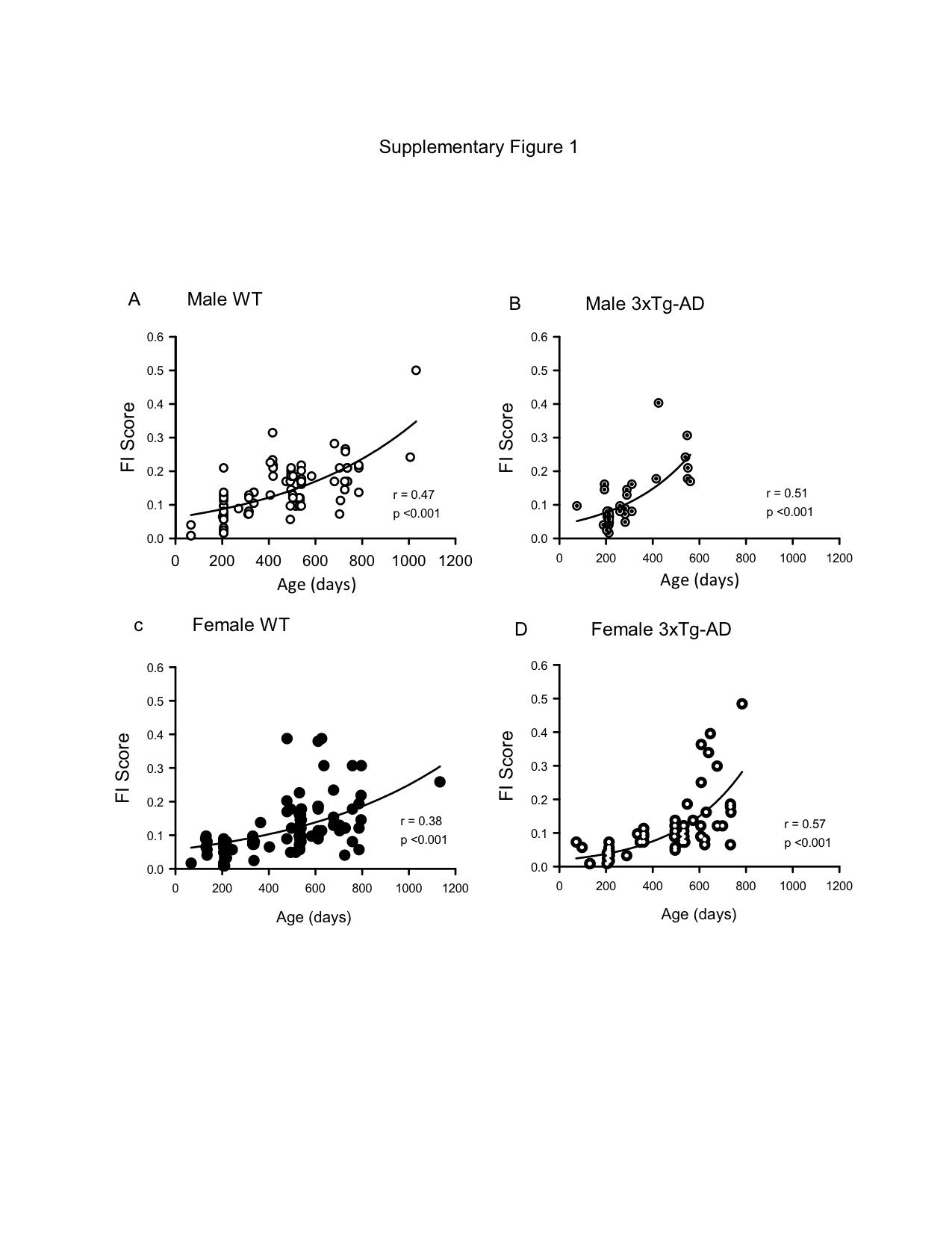

Supplement: FIGURE S1 — Increasing FI scores are correlated with increasing age in male and female 3xTg-AD and WT mice. FI Score is exponentially correlated with age (days) for (A) WT males (n = 84, r = 0.474, p < 0.0001), (B) 3xTg-AD males (n = 35, r = 0.461, p < 0.0001), (C) WT females (n = 91, r = 0.377, p < 0.0001) and (D) 3xTG females (n = 62, r = 0.574, p < 0.0001). [file Image_1.JPEG]
