# Supplementary material for: Sex Differences in Healthspan Predict Lifespan in the 3xTg-AD Mouse Model of Alzheimer’s Disease
Source: Front Aging Neurosci. 2018 Jun 12;10:172. doi: 10.3389/fnagi.2018.00172 (PMC6005856; doi:10.3389/fnagi.2018.00172)
Supplement: TABLE S1 — Causes of death or reason for euthanasia in WT and 3xTg-AD mice of both sexes. Numbers represent the number of mice in each category. Most mice were used in other experiments. [file Data_Sheet_1.docx]

**Supplemental Table 1: Causes of death or reason for euthanasia**

| **CAUSES^1^** | **MALE** | | **FEMALE** | |
| --- | --- | --- | --- | --- |
|  | **WT** | **3xTg-AD** | **WT** | **3xTg-AD** |
| Found dead | 6 | 7 | 9 | 8 |
| Enlarged spleen | 1 | 3 | 0 | 1 |
| Excessive grooming | 1 | 0 | 2 | 0 |
| Lesions | 0 | 0 | 1 | 0 |
| Obesity | 0 | 0 | 1 | 0 |
| Paralysis | 1 | 0 | 0 | 0 |
| Tumors | 1 | 0 | 0 | 0 |
| Penile/uterine prolapse | 1 | 0 | 0 | 0 |
| Anal prolapse | 0 | 1 | 0 | 0 |

^1^ Causes of death or reason for euthanasia in WT and 3xTg-AD mice of both sexes. Numbers represent the number of mice in each category. Most mice were used in other experiments.

**Supplemental Table 2: Raw data used to construct a frailty index from wild-type and 3xTg-AD mice of both sexes**

| **Mouse ID** | **Sex (female = 1, male =2)** | **Genotype (WT = 1, TG =2)** | **age (days) at FI** | **FI** | **age (days) at death** | **censored** | **Frailty Index Components** | | | | | | | | |
| --- | --- | --- | --- | --- | --- | --- | --- | --- | --- | --- | --- | --- | --- | --- | --- |
|  |  |  |  |  |  |  | **integument score** | **muscular-skeletal score** | **vestibular system score** | **ocular system score** | **digestive system score** | **respiratory system score** | **discomfort score** | **temperature score** | **weight score** |
| 2324 | 1 | 2 | 131 | 0.01 | 132 | 1 | 0 | 0 | 0 | 0 | 0 | 0 | 0 | 0 | 0 |
| 2325 | 1 | 2 | 131 | 0.01 | 132 | 1 | 0 | 0 | 0 | 0 | 0 | 0 | 0 | 0 | 0 |
| 2356 | 1 | 2 | 97 | 0.06 | 98 | 1 | 0 | 0.5 | 0 | 0.5 | 0 | 0 | 0 | 0 | 1 |
| 2966 | 1 | 2 | 213 | 0.06 | 296 | 1 | 0.5 | 0 | 0.5 | 0.5 | 0 | 0 | 0 | 0 | 0 |
| 2967 | 1 | 2 | 213 | 0.03 | 296 | 1 | 0.5 | 0.5 | 0 | 0 | 0 | 0 | 0 | 0 | 0 |
| 2968 | 1 | 2 | 213 | 0.07 | 296 | 1 | 0 | 0.5 | 0.5 | 1 | 0 | 0 | 0 | 0 | 0 |
| 2969 | 1 | 2 | 213 | 0.05 | 296 | 1 | 0.5 | 0.5 | 0 | 0 | 0 | 0 | 0 | 0 | 1 |
| 3014 | 1 | 2 | 204 | 0.01 | 287 | 1 | 0 | 0 | 0 | 0 | 0 | 0 | 0 | 0 | 0 |
| 3017 | 1 | 2 | 212 | 0.02 | 295 | 1 | 0 | 0.5 | 0 | 0 | 0 | 0 | 0 | 0 | 0 |
| 3018 | 1 | 2 | 212 | 0.05 | 295 | 1 | 0 | 0.5 | 0 | 0 | 0 | 0.5 | 0 | 0 | 0 |
| 3023 | 1 | 2 | 214 | 0.02 | 295 | 1 | 0 | 0.5 | 0 | 0 | 0 | 0 | 0 | 0 | 0 |
| 3024 | 1 | 2 | 214 | 0.04 | 295 | 1 | 0 | 1 | 0 | 0 | 0 | 0 | 0 | 0 | 0 |
| 3025 | 1 | 2 | 214 | 0.03 | 295 | 1 | 0.5 | 0 | 0 | 0 | 0 | 0 | 0.5 | 0 | 0 |
| 3032 | 1 | 2 | 205 | 0 | 286 | 1 | 0 | 0 | 0 | 0 | 0 | 0 | 0 | 0 | 0 |
| 3033 | 1 | 2 | 206 | 0.02 | 287 | 1 | 0 | 0.5 | 0 | 0 | 0 | 0 | 0 | 0 | 0 |
| 3068 | 1 | 2 | 72 | 0.07 | 497 | 0 | 1 | 0 | 0 | 0.5 | 0 | 0 | 0 | 0 | 1 |
| 3070 | 1 | 2 | 288 | 0.03 | 581 | 1 | 0.5 | 0.5 | 0 | 0 | 0 | 0 | 0 | 0 | 0 |
| 3417 | 1 | 2 | 205 | 0 |  |  | 0 | 0 | 0 | 0 | 0 | 0 | 0 | 0 | 0 |
| 3418 | 1 | 2 | 205 | 0.04 |  |  | 0 | 1 | 0 | 0 | 0 | 0 | 0 | 0 | 0 |
| 2162 | 1 | 2 | 347 | 0.07 | 654 | 1 | 0 | 0 | 0 | 1 | 0 | 0 | 1 | 0 | 0 |
| 2163 | 1 | 2 | 347 | 0.07 | 654 | 1 | 0 | 1 | 0 | 0.5 | 0 | 0 | 0.5 | 0 | 0 |
| 2603 | 1 | 2 | 526 | 0.07 | 540 | 1 | 1 | 0 | 0 | 0 | 0 | 0.5 | 0 | 0 | 1 |
| 2604 | 1 | 2 | 526 | 0.1 | 540 | 1 | 1.5 | 0.5 | 0 | 0 | 0 | 0.5 | 0 | 0 | 1 |
| 2610 | 1 | 2 | 526 | 0.08 |  |  | 1.5 | 1 | 0 | 0 | 0 | 0 | 0 | 0 | 0 |
| 2612 | 1 | 2 | 496 | 0.05 | 550 | 1 | 1.5 | 0 | 0 | 0 | 0 | 0 | 0 | 0 | 0 |
| 2703 | 1 | 2 | 335 | 0.1 | 353 | 0 | 1 | 0.5 | 0 | 1 | 0 | 0 | 0 | 0 | 0 |
| 2705 | 1 | 2 | 548 | 0.19 |  |  | 2 | 2.5 | 0 | 0 | 0 | 0.5 | 0 | 0 | 1 |
| 2747 | 1 | 2 | 496 | 0.1 |  |  | 2.5 | 0.5 | 0 | 0 | 0 | 0 | 0 | 0 | 0 |
| 2748 | 1 | 2 | 495 | 0.12 |  |  | 2.5 | 1 | 0 | 0 | 0 | 0 | 0 | 0 | 0 |
| 2749 | 1 | 2 | 496 | 0.14 |  |  | 2.5 | 0.5 | 0 | 0.5 | 0 | 0 | 0 | 0 | 1 |
| 2750 | 1 | 2 | 496 | 0.06 |  |  | 1.5 | 0 | 0 | 0 | 0 | 0 | 0 | 0 | 0 |
| 2789 | 1 | 2 | 493 | 0.09 | 620 | 0 | 1.5 | 1 | 0 | 0 | 0 | 0 | 0 | 0 | 0 |
| 2805 | 1 | 2 | 361 | 0.11 | 368 | 1 | 1 | 1 | 0.5 | 0 | 0 | 0.5 | 0 | 0 | 0 |
| 2806 | 1 | 2 | 361 | 0.07 | 368 | 1 | 1.5 | 0.5 | 0 | 0 | 0 | 0 | 0 | 0 | 0 |
| 2809 | 1 | 2 | 361 | 0.1 | 376 | 1 | 1 | 1.5 | 0 | 0 | 0 | 0 | 0 | 0 | 0 |
| 2810 | 1 | 2 | 361 | 0.09 | 376 | 1 | 1 | 1.5 | 0 | 0 | 0 | 0 | 0 | 0 | 0 |
| 2937 | 1 | 2 | 533 | 0.12 | 677 | 1 | 1.5 | 1 | 0 | 0 | 0 | 0 | 0 | 0 | 1 |
| 2938 | 1 | 2 | 533 | 0.12 | 677 | 1 | 2 | 0.5 | 0 | 0 | 0 | 0.5 | 0.5 | 0 | 0 |
| 2939 | 1 | 2 | 533 | 0.1 | 677 | 1 | 0.5 | 1.5 | 1 | 0 | 0 | 0 | 0 | 0 | 0 |
| 3069 | 1 | 2 | 573 | 0.14 | 581 | 1 | 1.5 | 1.5 | 0 | 0 | 0 | 0.5 | 0.5 | 0 | 0 |
| 3077 | 1 | 2 | 537 | 0.07 | 542 | 1 | 0.5 | 1.5 | 0 | 0 | 0 | 0 | 0 | 0 | 0 |
| 3078 | 1 | 2 | 537 | 0.07 | 542 | 1 | 1 | 1 | 0 | 0 | 0 | 0 | 0 | 0 | 0 |
| 3083 | 1 | 2 | 534 | 0.09 | 582 | 1 | 1 | 1.5 | 0 | 0 | 0 | 0 | 0 | 0 | 0 |
| 3086 | 1 | 2 | 532 | 0.12 | 582 | 0 | 1 | 1 | 1 | 0.5 | 0 | 0 | 0 | 0 | 0 |
| 1551 | 1 | 2 | 733 | 0.06 | 733 | 1 | 0 | 2 | 0 | 0 | 0 | 0 | 0 | 0 | 0 |
| 1552 | 1 | 2 | 733 | 0.18 | 733 | 1 | 0 | 4 | 0.5 | 1 | 0 | 0 | 0 | 0 | 0 |
| 1553 | 1 | 2 | 733 | 0.19 | 733 | 1 | 2.5 | 1.5 | 0 | 1.5 | 0 | 0 | 0 | 0 | 0 |
| 2224 | 1 | 2 | 699 | 0.12 | 699 | 1 | 1.5 | 1 | 1 | 0 | 0 | 0 | 0 | 0 | 0 |
| 2322 | 1 | 2 | 677 | 0.12 | 702 | 1 | 1.5 | 1 | 0.5 | 0.5 | 0 | 0 | 0 | 0 | 0 |
| 2432 | 1 | 2 | 623 | 0.08 | 647 | 1 | 1 | 0.5 | 0 | 0.5 | 0 | 0 | 0 | 1 | 0 |
| 2433 | 1 | 2 | 623 | 0.06 | 647 | 1 | 1 | 0.5 | 0 | 0 | 0 | 0 | 0 | 1 | 0 |
| 2621 | 1 | 2 | 608 | 0.09 | 631 | 0 | 1.5 | 0.5 | 0 | 0.5 | 0 | 0 | 0 | 0 | 0 |
| 2807 | 1 | 2 | 735 | 0.16 | 749 | 1 | 2 | 1 | 1 | 0 | 0 | 0.5 | 0 | 1 | 0 |
| 2808 | 1 | 2 | 629 | 0.16 | 714 | 0 | 1 | 2 | 1 | 0.5 | 0 | 0 | 0 | 1 | 0 |
| 2862 | 1 | 2 | 606 | 0.09 | 620 | 1 | 1 | 1 | 0 | 0 | 0 | 0.5 | 0 | 0 | 0 |
| 2863 | 1 | 2 | 606 | 0.12 | 620 | 1 | 1.5 | 2 | 0 | 0 | 0 | 0 | 0 | 0 | 0 |
| 2312 | 1 | 2 | 676 | 0.3 | 684 | 0 | 3 | 5 | 0 | 0 | 0 | 1 | 0 | 0 | 0 |
| 2313 | 1 | 2 | 639 | 0.34 | 914 | 1 | 2 | 4.5 | 1 | 1.5 | 0 | 1 | 0 | 1 | 0 |
| 2618 | 1 | 2 | 608 | 0.36 | 672 | 0 | 3.5 | 4.5 | 1 | 0.5 | 0 | 1 | 0.5 | 0 | 0 |
| 2619 | 1 | 2 | 783 | 0.48 | 785 | 1 | 3 | 7 | 2 | 2 | 0 | 1 | 0 | 0 | 0 |
| 2620 | 1 | 2 | 608 | 0.25 | 763 | 1 | 2.5 | 3.5 | 0 | 0 | 0 | 1 | 0.5 | 0 | 0 |
| 2790 | 1 | 2 | 647 | 0.4 | 661 | 0 | 3 | 4.5 | 1 | 2 | 0.5 | 1 | 0 | 0 | 0 |
| 2296 | 1 | 1 | 131 | 0.06 | 132 | 1 | 0 | 0.5 | 0 | 1 | 0 | 0 | 0 | 0 | 1 |
| 2297 | 1 | 1 | 131 | 0.06 | 132 | 1 | 0.5 | 0 | 0 | 1.5 | 0 | 0 | 0 | 0 | 0 |
| 2298 | 1 | 1 | 131 | 0.1 | 132 | 1 | 0 | 0 | 1 | 1 | 0 | 0 | 0 | 0 | 1 |
| 2584 | 1 | 1 | 135 | 0.06 | 135 | 1 | 1 | 0 | 0 | 0.5 | 0 | 0 | 0 | 0 | 0 |
| 2585 | 1 | 1 | 135 | 0.08 | 135 | 1 | 1.5 | 0 | 0 | 1 | 0 | 0 | 0 | 0 | 0 |
| 2586 | 1 | 1 | 135 | 0.04 | 135 | 1 | 0.5 | 0 | 0 | 0.5 | 0 | 0 | 0 | 0 | 0 |
| 2943 | 1 | 1 | 129 | 0.09 | 676 | 1 | 0.5 | 0 | 1 | 1 | 0 | 0 | 0 | 0 | 0 |
| 2958 | 1 | 1 | 210 | 0.03 | 211 | 0 | 0 | 0 | 0 | 0.5 | 0 | 0 | 0 | 0 | 1 |
| 2959 | 1 | 1 | 210 | 0.01 | 212 | 0 | 0 | 0 | 0 | 0 | 0 | 0 | 0 | 0 | 0 |
| 2960 | 1 | 1 | 210 | 0 | 213 | 0 | 0 | 0 | 0 | 0 | 0 | 0 | 0 | 0 | 0 |
| 3034 | 1 | 1 | 206 | 0.05 | 287 | 1 | 1 | 0 | 0 | 0.5 | 0 | 0 | 0 | 0 | 0 |
| 3035 | 1 | 1 | 206 | 0.08 | 287 | 1 | 1 | 0 | 0 | 1.5 | 0 | 0 | 0 | 0 | 0 |
| 3036 | 1 | 1 | 206 | 0.06 | 287 | 1 | 1 | 0 | 0 | 1 | 0 | 0 | 0 | 0 | 0 |
| 3037 | 1 | 1 | 206 | 0.09 | 287 | 1 | 1 | 0 | 0 | 1.5 | 0 | 0 | 0 | 0 | 0 |
| 3048 | 1 | 1 | 206 | 0.06 | 287 | 1 | 0 | 1.5 | 0 | 0 | 0 | 0 | 0 | 1 | 0 |
| 3049 | 1 | 1 | 206 | 0 | 287 | 1 | 0 | 0 | 0 | 0 | 0 | 0 | 0 | 0 | 0 |
| 3050 | 1 | 1 | 206 | 0.01 | 328 | 1 | 0 | 0 | 0 | 0 | 0 | 0 | 0 | 0 | 0 |
| 3051 | 1 | 1 | 206 | 0.02 | 328 | 1 | 0 | 0.5 | 0 | 0 | 0 | 0 | 0 | 0 | 0 |
| 3090 | 1 | 1 | 243 | 0.06 | 271 | 0 | 0 | 0.5 | 0 | 0.5 | 0 | 0 | 0 | 1 | 0 |
| 3236 | 1 | 1 | 67 | 0.02 | 326 | 0 | 0.5 | 0 | 0 | 0 | 0 | 0 | 0 | 0 | 0 |
| 3344 | 1 | 1 | 218 | 0.08 |  |  | 1 | 0 | 0 | 1 | 0 | 0 | 0 | 0 | 1 |
| 3347 | 1 | 1 | 218 | 0.03 |  |  | 0 | 0 | 0 | 1 | 0 | 0 | 0 | 0 | 0 |
| 2169 | 1 | 1 | 336 | 0.07 | 643 | 1 | 0 | 1.5 | 0 | 0 | 0 | 0 | 0.5 | 0 | 0 |
| 2170 | 1 | 1 | 364 | 0.14 | 413 | 1 | 0 | 1.5 | 1 | 0.5 | 0 | 0 | 0.5 | 1 | 0 |
| 2187 | 1 | 1 | 337 | 0.08 | 644 | 1 | 0 | 1 | 0 | 0.5 | 0 | 0.5 | 0.5 | 0 | 0 |
| 2441 | 1 | 1 | 583 | 0.1 | 858 | 1 | 1.5 | 0.5 | 0.5 | 0.5 | 0 | 0 | 0 | 0 | 0 |
| 2682 | 1 | 1 | 477 | 0.2 | 484 | 1 | 1 | 2.5 | 1 | 0.5 | 0 | 0.5 | 0 | 1 | 0 |
| 2683 | 1 | 1 | 477 | 0.09 | 484 | 1 | 0 | 2 | 0 | 0.5 | 0 | 0 | 0 | 0 | 0 |
| 2690 | 1 | 1 | 531 | 0.16 | 556 | 1 | 1 | 1 | 1 | 1.5 | 0 | 0.5 | 0 | 0 | 0 |
| 2693 | 1 | 1 | 531 | 0.16 | 556 | 1 | 2.5 | 1 | 0.5 | 0.5 | 0 | 0.5 | 0 | 0 | 0 |
| 2793 | 1 | 1 | 493 | 0.18 | 689 | 0 | 2.5 | 0.5 | 0.5 | 1.5 | 0 | 0.5 | 0 | 0 | 0 |
| 2794 | 1 | 1 | 493 | 0.05 | 689 | 0 | 1 | 0 | 0 | 0.5 | 0 | 0 | 0 | 0 | 0 |
| 2860 | 1 | 1 | 497 | 0.12 | 497 | 1 | 0.5 | 1 | 1 | 0.5 | 0 | 0.5 | 0 | 0 | 0 |
| 2916 | 1 | 1 | 402 | 0.06 | 409 | 1 | 1.5 | 0.5 | 0 | 0 | 0 | 0 | 0 | 0 | 0 |
| 2917 | 1 | 1 | 539 | 0.15 | 558 | 1 | 2 | 0 | 1 | 1.5 | 0 | 0 | 0 | 0 | 0 |
| 2918 | 1 | 1 | 539 | 0.18 | 558 | 1 | 2.5 | 2 | 1 | 0 | 0 | 0 | 0 | 0 | 0 |
| 2919 | 1 | 1 | 539 | 0.18 | 542 | 0 | 1.5 | 1 | 2 | 0.5 | 0 | 0 | 0 | 0 | 0 |
| 2926 | 1 | 1 | 538 | 0.12 | 549 | 1 | 1 | 0.5 | 1 | 0.5 | 0.5 | 0 | 0 | 0 | 0 |
| 2927 | 1 | 1 | 538 | 0.08 | 549 | 1 | 0.5 | 1 | 0.5 | 0 | 0 | 0 | 0 | 0 | 0 |
| 2929 | 1 | 1 | 538 | 0.1 | 549 | 1 | 1 | 1.5 | 0 | 0.5 | 0 | 0 | 0 | 0 | 0 |
| 2944 | 1 | 1 | 536 | 0.1 | 676 | 1 | 1 | 0 | 0.5 | 1.5 | 0 | 0 | 0 | 0 | 0 |
| 2945 | 1 | 1 | 536 | 0.1 | 676 | 1 | 1 | 0.5 | 0.5 | 1 | 0 | 0 | 0 | 0 | 0 |
| 2946 | 1 | 1 | 536 | 0.16 | 676 | 1 | 1.5 | 1 | 1 | 1.5 | 0 | 0 | 0 | 0 | 0 |
| 2947 | 1 | 1 | 532 | 0.06 | 676 | 1 | 1 | 0 | 0 | 0 | 0 | 0.5 | 0 | 0 | 0 |
| 2954 | 1 | 1 | 534 | 0.17 | 549 | 1 | 1.5 | 0 | 1 | 1.5 | 0.5 | 0 | 0 | 0 | 1 |
| 2955 | 1 | 1 | 534 | 0.1 | 549 | 1 | 2 | 0.5 | 0 | 0.5 | 0 | 0 | 0 | 0 | 0 |
| 2956 | 1 | 1 | 534 | 0.15 | 549 | 1 | 2 | 1 | 1 | 0 | 0 | 0 | 0 | 1 | 0 |
| 2957 | 1 | 1 | 534 | 0.13 | 549 | 1 | 2 | 0.5 | 0 | 1.5 | 0 | 0 | 0 | 0 | 0 |
| 3102 | 1 | 1 | 531 | 0.07 | 581 | 1 | 0.5 | 0 | 1 | 0.5 | 0 | 0 | 0 | 0 | 0 |
| 3103 | 1 | 1 | 531 | 0.1 | 581 | 1 | 0 | 1 | 1 | 1 | 0 | 0 | 0 | 0 | 0 |
| 3104 | 1 | 1 | 531 | 0.06 |  |  | 0 | 0.5 | 1 | 0.5 | 0 | 0 | 0 | 0 | 0 |
| 3105 | 1 | 1 | 515 | 0.05 | 581 | 1 | 0 | 0 | 1 | 0.5 | 0 | 0 | 0 | 0 | 0 |
| 3111 | 1 | 1 | 533 | 0.09 | 581 | 1 | 0 | 1.5 | 0 | 1 | 0 | 0 | 0 | 0 | 0 |
| 3112 | 1 | 1 | 533 | 0.09 | 581 | 1 | 0 | 2 | 0 | 0.5 | 0 | 0 | 0 | 0 | 0 |
| 3123 | 1 | 1 | 533 | 0.09 | 584 | 1 | 0.5 | 0.5 | 1 | 0.5 | 0 | 0 | 0 | 0 | 0 |
| 3170 | 1 | 1 | 336 | 0.02 | 537 | 1 | 0 | 0 | 0 | 0 | 0 | 0 | 0 | 0 | 1 |
| 3237 | 1 | 1 | 331 | 0.1 | 537 | 1 | 0.5 | 0.5 | 0 | 1.5 | 0 | 0 | 0 | 0 | 1 |
| 3238 | 1 | 1 | 331 | 0.07 | 537 | 1 | 1 | 0 | 0 | 1 | 0 | 0 | 0 | 0 | 0 |
| 3239 | 1 | 1 | 331 | 0.08 | 537 | 1 | 1 | 0 | 0 | 1.5 | 0 | 0 | 0 | 0 | 0 |
| 2691 | 1 | 1 | 478 | 0.39 | 519 | 0 | 3 | 5 | 1.5 | 1 | 0 | 1 | 0 | 1 | 0 |
| 2692 | 1 | 1 | 531 | 0.23 | 556 | 1 | 2.5 | 1.5 | 1.5 | 1 | 0 | 0.5 | 0 | 0 | 0 |
| 2822 | 2 | 1 | 611 | 0.19 | 612 | 1 | 0.5 | 2.5 | 1 | 1.5 | 0 | 0 | 0 | 0 | 0 |
| 3147 | 2 | 1 | 530 | 0.1 |  |  | 0.5 | 2 | 0 | 0 | 0 | 0.5 | 0 | 0 | 0 |
| 2821 | 2 | 1 | 611 | 0.38 | 612 | 1 | 3.5 | 3.5 | 2 | 2 | 0.5 | 0 | 0 | 0 | 0 |
| 1517 | 1 | 1 | 758 | 0.08 | 758 | 1 | 0 | 0 | 1 | 1 | 0 | 0 | 0 | 1 | 0 |
| 1518 | 1 | 1 | 758 | 0.18 | 758 | 1 | 0 | 2 | 1 | 2.5 | 0 | 0 | 0 | 0 | 0 |
| 2283 | 1 | 1 | 681 | 0.14 | 703 | 1 | 1.5 | 1 | 1 | 0.5 | 0 | 0 | 0 | 0 | 0 |
| 2288 | 1 | 1 | 677 | 0.15 | 702 | 1 | 2 | 0.5 | 1 | 0.5 | 0 | 0.5 | 0 | 0 | 0 |
| 2289 | 1 | 1 | 677 | 0.13 | 701 | 1 | 1.5 | 0.5 | 1 | 0.5 | 0 | 0.5 | 0 | 0 | 0 |
| 2370 | 1 | 1 | 626 | 0.11 | 650 | 1 | 2 | 0 | 1 | 0.5 | 0 | 0 | 0 | 0 | 0 |
| 2439 | 1 | 1 | 703 | 0.11 |  |  | 0.5 | 2 | 0 | 0.5 | 0 | 0 | 0 | 1 | 0 |
| 2440 | 1 | 1 | 703 | 0.13 |  |  | 2 | 0.5 | 0 | 1.5 | 0 | 0 | 0 | 0 | 0 |
| 2460 | 1 | 1 | 725 | 0.04 |  |  | 0.5 | 0 | 0 | 0 | 0 | 0.5 | 0 | 0 | 0 |
| 2739 | 1 | 1 | 795 | 0.15 | 801 | 1 | 0.5 | 0.5 | 1 | 1.5 | 0.5 | 0 | 0 | 1 | 0 |
| 2755 | 1 | 1 | 786 | 0.06 | 791 | 1 | 0 | 0.5 | 1 | 0 | 0 | 0 | 0 | 0 | 0 |
| 2757 | 1 | 1 | 786 | 0.19 | 791 | 1 | 2 | 1 | 1 | 2 | 0 | 0 | 0 | 0 | 0 |
| 2758 | 1 | 1 | 786 | 0.12 | 791 | 1 | 1.5 | 0.5 | 1 | 0.5 | 0 | 0 | 0 | 0 | 0 |
| 2815 | 1 | 1 | 611 | 0.11 | 612 | 1 | 2 | 0 | 1 | 0.5 | 0 | 0 | 0 | 0 | 0 |
| 2816 | 1 | 1 | 611 | 0.1 | 612 | 1 | 0.5 | 1 | 1 | 0.5 | 0 | 0 | 0 | 0 | 0 |
| 2817 | 1 | 1 | 726 | 0.12 | 732 | 1 | 1.5 | 0.5 | 1 | 0.5 | 0 | 0 | 0 | 0 | 0 |
| 2817 | 1 | 1 | 611 | 0.19 | 732 | 1 | 1.5 | 1 | 1 | 1.5 | 0.5 | 0 | 0 | 0 | 0 |
| 2818 | 1 | 1 | 611 | 0.09 | 743 | 1 | 0.5 | 0.5 | 1 | 0.5 | 0 | 0 | 0 | 0 | 0 |
| 2819 | 1 | 1 | 727 | 0.12 | 743 | 1 | 2 | 0.5 | 0.5 | 0.5 | 0 | 0 | 0 | 0 | 0 |
| 2820 | 1 | 1 | 611 | 0.18 | 694 | 1 | 1 | 1.5 | 1 | 2 | 0 | 0 | 0 | 0 | 0 |
| 1452 | 1 | 1 | 796 | 0.31 | 796 | 1 | 2.5 | 2.5 | 1 | 2.5 | 0 | 0.5 | 0 | 1 | 0 |
| 1516 | 1 | 1 | 758 | 0.31 | 758 | 1 | 1 | 4 | 1 | 2 | 0.5 | 0.5 | 0 | 1 | 0 |
| 1794 | 1 | 1 | 1133 | 0.26 | 1193 | 0 | 2 | 2.5 | 1 | 1.5 | 0 | 0.5 | 0 | 1 | 0 |
| 2287 | 1 | 1 | 677 | 0.23 | 701 | 1 | 3 | 3.5 | 0.5 | 0 | 0 | 0 | 0 | 0 | 0 |
| 2369 | 1 | 1 | 626 | 0.39 | 650 | 1 | 2.5 | 5 | 0.5 | 2 | 0.5 | 1 | 0 | 1 | 0 |
| 2740 | 1 | 1 | 795 | 0.22 | 801 | 1 | 2 | 1.5 | 1.5 | 1 | 0.5 | 0 | 0 | 0 | 0 |
| 2756 | 1 | 1 | 636 | 0.31 | 680 | 0 | 3.5 | 2.5 | 0 | 2 | 0 | 0.5 | 0.5 | 1 | 0 |
| 2941 | 2 | 2 | 213 | 0.06 | 229 | 0 | 1.5 | 0 | 0 | 0 | 0 | 0 | 0 | 0 | 0 |
| 2970 | 2 | 2 | 213 | 0.07 | 289 | 1 | 1.5 | 0.5 | 0 | 0 | 0 | 0 | 0 | 0 | 0 |
| 2971 | 2 | 2 | 203 | 0.02 | 203 | 0 | 0.5 | 0 | 0 | 0 | 0 | 0 | 0 | 0 | 0 |
| 2972 | 2 | 2 | 213 | 0.05 | 289 | 1 | 0.5 | 0.5 | 0 | 0.5 | 0 | 0 | 0 | 0 | 0 |
| 2973 | 2 | 2 | 213 | 0.06 | 213 | 1 | 0.5 | 0 | 0 | 1.5 | 0 | 0 | 0 | 0 | 0 |
| 3013 | 2 | 2 | 204 | 0.08 | 280 | 1 | 1 | 0.5 | 0 | 1 | 0 | 0 | 0 | 0 | 0 |
| 3015 | 2 | 2 | 212 | 0.04 | 288 | 1 | 0.5 | 0.5 | 0 | 0 | 0 | 0 | 0 | 0 | 0 |
| 3016 | 2 | 2 | 212 | 0.02 | 288 | 1 | 0.5 | 0 | 0 | 0 | 0 | 0 | 0 | 0 | 0 |
| 3020 | 2 | 2 | 212 | 0.08 | 288 | 1 | 1.5 | 0.5 | 0 | 0.5 | 0 | 0 | 0 | 0 | 0 |
| 3021 | 2 | 2 | 212 | 0.06 | 288 | 1 | 0.5 | 0.5 | 0 | 0.5 | 0 | 0 | 0 | 0 | 0 |
| 3026 | 2 | 2 | 214 | 0.06 | 288 | 1 | 1 | 0 | 0 | 0 | 0 | 0.5 | 0.5 | 0 | 0 |
| 3028 | 2 | 2 | 205 | 0.06 | 279 | 1 | 1.5 | 0.5 | 0 | 0 | 0 | 0 | 0 | 0 | 0 |
| 3029 | 2 | 2 | 205 | 0.08 | 279 | 1 | 1.5 | 0 | 0 | 0.5 | 0 | 0 | 0 | 0 | 0 |
| 3030 | 2 | 2 | 205 | 0.03 | 279 | 1 | 1 | 0 | 0 | 0 | 0 | 0 | 0 | 0 | 0 |
| 3066 | 2 | 2 | 75 | 0.1 | 129 | 1 | 2 | 0 | 0 | 0 | 0 | 0 | 0.5 | 0 | 0 |
| 3088 | 2 | 2 | 289 | 0.13 | 447 | 0 | 1 | 1 | 1.5 | 0 | 0.5 | 0 | 0 | 0 | 0 |
| 3089 | 2 | 2 | 289 | 0.15 | 343 | 0 | 1 | 2 | 0 | 0 | 0.5 | 0 | 0.5 | 0 | 0 |
| 3278 | 2 | 2 | 260 | 0.1 | 483 | 1 | 1 | 1.5 | 0 | 0.5 | 0 | 0 | 0 | 0 | 0 |
| 3279 | 2 | 2 | 260 | 0.08 | 481 | 0 | 0.5 | 1 | 0 | 1 | 0 | 0 | 0 | 0 | 0 |
| 3282 | 2 | 2 | 282 | 0.07 | 377 | 1 | 1.5 | 0.5 | 0 | 0 | 0 | 0 | 0 | 0 | 0 |
| 3283 | 2 | 2 | 282 | 0.09 | 378 | 0 | 1 | 1 | 0 | 0.5 | 0 | 0 | 0 | 0 | 0 |
| 3284 | 2 | 2 | 282 | 0.05 | 523 | 0 | 1 | 0 | 0 | 0.5 | 0 | 0 | 0 | 0 | 0 |
| 3340 | 2 | 2 | 272 | 0.09 | 499 | 1 | 1 | 1 | 0 | 0 | 0 | 0 | 0 | 0 | 1 |
| 3415 | 2 | 2 | 193 | 0.15 |  |  | 2.5 | 0.5 | 0 | 0.5 | 0 | 0.5 | 0 | 0 | 0 |
| 3416 | 2 | 2 | 193 | 0.16 | 202 | 0 | 2.5 | 0.5 | 0 | 0.5 | 1 | 0.5 | 0 | 0 | 0 |
| 3434 | 2 | 2 | 189 | 0.04 | 516 | 1 | 0 | 0 | 0 | 0.5 | 0 | 0 | 0 | 0 | 1 |
| 2459 | 2 | 2 | 561 | 0.17 | 836 | 1 | 2 | 1.5 | 0.5 | 0.5 | 0.5 | 0 | 0 | 0 | 0 |
| 2813 | 2 | 2 | 415 | 0.18 | 599 | 1 | 2 | 2 | 0 | 0 | 0.5 | 0.5 | 0 | 0 | 1 |
| 3067 | 2 | 2 | 550 | 0.18 |  |  | 1 | 2 | 1 | 0.5 | 0 | 0.5 | 0 | 0 | 0 |
| 3079 | 2 | 2 | 551 | 0.21 | 615 | 0 | 2.5 | 3.5 | 0 | 0 | 0 | 0 | 0 | 0 | 0 |
| 3195 | 2 | 2 | 311 | 0.16 | 428 | 1 | 1.5 | 2 | 0 | 1 | 0 | 0 | 0 | 0 | 1 |
| 3196 | 2 | 2 | 311 | 0.08 | 322 | 0 | 1.5 | 0.5 | 0 | 0 | 0 | 0 | 0 | 0 | 1 |
| 2940 | 2 | 2 | 425 | 0.4 | 683 | 1 | 2.5 | 5 | 0 | 0.5 | 1 | 1 | 1.5 | 0 | 1 |
| 3075 | 2 | 2 | 540 | 0.24 | 540 | 1 | 2 | 3 | 0.5 | 0 | 1 | 0.5 | 0 | 0 | 0 |
| 3087 | 2 | 2 | 548 | 0.31 | 588 | 0 | 3.5 | 3 | 0 | 2 | 0 | 0.5 | 0 | 0 | 0 |
| 2864 | 2 | 1 | 270 | 0.09 | 610 | 1 | 1.5 | 1 | 0 | 0 | 0 | 0 | 0 | 0 | 0 |
| 3038 | 2 | 1 | 206 | 0.02 | 280 | 1 | 0 | 0.5 | 0 | 0 | 0 | 0 | 0 | 0 | 0 |
| 3039 | 2 | 1 | 206 | 0.06 | 280 | 1 | 0.5 | 0 | 0 | 1 | 0 | 0 | 0 | 0 | 0 |
| 3040 | 2 | 1 | 206 | 0.02 | 280 | 1 | 0 | 0.5 | 0 | 0 | 0 | 0 | 0 | 0 | 0 |
| 3041 | 2 | 1 | 206 | 0.02 | 280 | 1 | 0.5 | 0 | 0 | 0 | 0 | 0 | 0 | 0 | 0 |
| 3042 | 2 | 1 | 206 | 0.14 | 280 | 1 | 0.5 | 1.5 | 1 | 1 | 0 | 0 | 0 | 0 | 0 |
| 3043 | 2 | 1 | 206 | 0.12 | 280 | 1 | 1.5 | 1 | 0 | 1 | 0 | 0 | 0 | 0 | 0 |
| 3044 | 2 | 1 | 206 | 0.08 | 280 | 1 | 1.5 | 1 | 0 | 0 | 0 | 0 | 0 | 0 | 0 |
| 3045 | 2 | 1 | 206 | 0.06 | 280 | 1 | 0.5 | 1.5 | 0 | 0 | 0 | 0 | 0 | 0 | 0 |
| 3046 | 2 | 1 | 206 | 0.06 | 280 | 1 | 0.5 | 0.5 | 0 | 0.5 | 0 | 0 | 0 | 0 | 0 |
| 3047 | 2 | 1 | 206 | 0.07 | 280 | 1 | 0.5 | 1.5 | 0 | 0 | 0 | 0 | 0 | 0 | 0 |
| 3052 | 2 | 1 | 206 | 0.03 | 280 | 1 | 1 | 0 | 0 | 0 | 0 | 0 | 0 | 0 | 0 |
| 3053 | 2 | 1 | 206 | 0.21 | 280 | 1 | 2.5 | 2 | 0 | 1 | 0 | 0.5 | 0 | 1 | 0 |
| 3054 | 2 | 1 | 206 | 0.13 |  |  | 1.5 | 2 | 0 | 0.5 | 0 | 0 | 0 | 0 | 0 |
| 3055 | 2 | 1 | 206 | 0.11 | 482 | 1 | 0 | 1 | 1 | 1.5 | 0 | 0 | 0 | 0 | 0 |
| 3056 | 2 | 1 | 206 | 0.1 | 482 | 1 | 1.5 | 0 | 0 | 1.5 | 0 | 0 | 0 | 0 | 0 |
| 3060 | 2 | 1 | 206 | 0.02 | 482 | 1 | 0 | 0 | 0 | 0.5 | 0 | 0 | 0 | 0 | 0 |
| 3061 | 2 | 1 | 206 | 0.02 | 482 | 1 | 0.5 | 0 | 0 | 0 | 0 | 0 | 0 | 0 | 0 |
| 3062 | 2 | 1 | 206 | 0.1 | 300 | 0 | 1.5 | 0.5 | 0 | 0 | 0 | 0.5 | 0 | 0 | 0 |
| 3242 | 2 | 1 | 66 | 0.01 | 658 | 1 | 0 | 0 | 0 | 0 | 0 | 0 | 0 | 0 | 0 |
| 3243 | 2 | 1 | 66 | 0.01 |  |  | 0 | 0 | 0 | 0 | 0 | 0 | 0 | 0 | 0 |
| 3244 | 2 | 1 | 66 | 0.04 | 617 | 0 | 0 | 0.5 | 0 | 0 | 0 | 0 | 0 | 0 | 1 |
| 3366 | 2 | 1 | 202 | 0.06 |  |  | 1 | 0 | 0 | 1 | 0 | 0 | 0 | 0 | 0 |
| 2435 | 2 | 1 | 583 | 0.19 | 679 | 1 | 1 | 1 | 1 | 1.5 | 1 | 0 | 0 | 0 | 0 |
| 2742 | 2 | 1 | 503 | 0.12 | 511 | 0 | 1 | 0.5 | 1 | 1 | 0 | 0 | 0 | 0 | 0 |
| 2744 | 2 | 1 | 504 | 0.19 | 557 | 0 | 4 | 0 | 0.5 | 0.5 | 0 | 0.5 | 0 | 0 | 0 |
| 2745 | 2 | 1 | 503 | 0.13 | 525 | 1 | 0.5 | 1.5 | 1 | 1 | 0 | 0 | 0 | 0 | 0 |
| 2759 | 2 | 1 | 495 | 0.19 | 523 | 1 | 2 | 2.5 | 0 | 1 | 0 | 0 | 0 | 0 | 0 |
| 2760 | 2 | 1 | 495 | 0.21 | 523 | 1 | 2 | 2.5 | 0 | 2 | 0 | 0 | 0 | 0 | 0 |
| 2761 | 2 | 1 | 495 | 0.19 | 523 | 1 | 2.5 | 2.5 | 0 | 1 | 0 | 0 | 0 | 0 | 0 |
| 2762 | 2 | 1 | 495 | 0.2 | 523 | 1 | 2.5 | 2 | 0.5 | 1 | 0 | 0 | 0 | 0 | 0 |
| 2763 | 2 | 1 | 495 | 0.15 | 523 | 1 | 2.5 | 1 | 0.5 | 0.5 | 0 | 0 | 0 | 0 | 0 |
| 2795 | 2 | 1 | 492 | 0.06 | 521 | 1 | 0 | 0.5 | 0.5 | 0.5 | 0 | 0 | 0 | 0 | 0 |
| 2796 | 2 | 1 | 492 | 0.17 | 521 | 1 | 2 | 0.5 | 1 | 1.5 | 0 | 0 | 0 | 0 | 0 |
| 2797 | 2 | 1 | 492 | 0.1 | 521 | 1 | 1 | 0 | 1 | 0.5 | 0 | 0 | 0 | 0 | 1 |
| 2861 | 2 | 1 | 474 | 0.17 | 497 | 1 | 2.5 | 2 | 0 | 0.5 | 0 | 0 | 0 | 0 | 0 |
| 2920 | 2 | 1 | 539 | 0.1 | 557 | 1 | 2.5 | 0 | 0 | 0.5 | 0 | 0 | 0 | 0 | 0 |
| 2921 | 2 | 1 | 539 | 0.2 | 557 | 1 | 2 | 2.5 | 0 | 1.5 | 0 | 0 | 0 | 0 | 0 |
| 2922 | 2 | 1 | 539 | 0.17 | 557 | 1 | 2 | 0.5 | 1 | 1.5 | 0 | 0 | 0 | 0 | 0 |
| 2923 | 2 | 1 | 540 | 0.17 | 550 | 1 | 2 | 0 | 1 | 2 | 0 | 0 | 0 | 0 | 0 |
| 2924 | 2 | 1 | 540 | 0.16 | 550 | 1 | 2.5 | 0.5 | 1 | 1 | 0 | 0 | 0 | 0 | 0 |
| 2925 | 2 | 1 | 540 | 0.18 | 550 | 1 | 3 | 0 | 1 | 1.5 | 0 | 0 | 0 | 0 | 0 |
| 2934 | 2 | 1 | 539 | 0.17 | 682 | 1 | 2.5 | 0 | 1 | 1.5 | 0 | 0 | 0 | 0 | 0 |
| 2951 | 2 | 1 | 418 | 0.19 | 418 | 1 | 1 | 2.5 | 1 | 1 | 0 | 0 | 0 | 0 | 0 |
| 2952 | 2 | 1 | 418 | 0.21 | 418 | 1 | 2 | 1 | 1.5 | 1.5 | 0 | 0 | 0 | 0 | 0 |
| 2961 | 2 | 1 | 530 | 0.1 | 547 | 1 | 1 | 1 | 0 | 0.5 | 0 | 0 | 0 | 0 | 0 |
| 2962 | 2 | 1 | 530 | 0.19 | 547 | 0 | 1 | 1 | 2 | 1.5 | 0 | 0 | 0 | 0 | 0 |
| 2963 | 2 | 1 | 530 | 0.12 | 547 | 0 | 1 | 1.5 | 0.5 | 0.5 | 0 | 0 | 0 | 0 | 0 |
| 3096 | 2 | 1 | 515 | 0.12 | 610 | 1 | 2 | 0.5 | 0 | 1 | 0 | 0 | 0 | 0 | 0 |
| 3097 | 2 | 1 | 515 | 0.1 | 685 | 1 | 1 | 0.5 | 0 | 1.5 | 0 | 0 | 0 | 0 | 0 |
| 3098 | 2 | 1 | 533 | 0.11 | 685 | 1 | 2 | 1 | 0 | 0.5 | 0 | 0 | 0 | 0 | 0 |
| 3099 | 2 | 1 | 515 | 0.16 | 685 | 1 | 1.5 | 2 | 0 | 1.5 | 0 | 0 | 0 | 0 | 0 |
| 3100 | 2 | 1 | 533 | 0.12 |  |  | 1 | 0.5 | 1 | 0.5 | 0 | 0 | 0 | 0 | 1 |
| 3106 | 2 | 1 | 515 | 0.1 | 842 | 0 | 1 | 2 | 0 | 0 | 0 | 0 | 0 | 0 | 0 |
| 3113 | 2 | 1 | 515 | 0.11 | 728 | 1 | 0.5 | 1.5 | 0.5 | 0.5 | 0.5 | 0 | 0 | 0 | 0 |
| 3175 | 2 | 1 | 314 | 0.12 | 663 | 1 | 1.5 | 0 | 1 | 1 | 0 | 0 | 0 | 0 | 0 |
| 3176 | 2 | 1 | 314 | 0.07 | 520 | 0 | 0.5 | 0.5 | 1 | 0 | 0 | 0 | 0 | 0 | 0 |
| 3177 | 2 | 1 |  | 0.13 | 663 | 1 | 0.5 | 1 | 0 | 1.5 | 0 | 0 | 0 | 1 | 1 |
| 3178 | 2 | 1 | 314 | 0.08 | 663 | 1 | 0.5 | 0 | 1 | 0.5 | 0 | 0 | 0 | 0 | 1 |
| 3179 | 2 | 1 | 314 | 0.08 | 663 | 1 | 0.5 | 1 | 0.5 | 0 | 0 | 0 | 0 | 0 | 1 |
| 3182 | 2 | 1 | 336 | 0.1 |  |  | 1 | 1 | 0 | 0.5 | 0 | 0 | 0.5 | 0 | 0 |
| 3184 | 2 | 1 | 336 | 0.14 | 541 | 0 | 1.5 | 1.5 | 0 | 0.5 | 0 | 0 | 0 | 0 | 1 |
| 3185 | 2 | 1 | 336 | 0.14 | 663 | 1 | 1.5 | 1.5 | 0 | 1 | 0 | 0 | 0 | 0 | 0 |
| 3192 | 2 | 1 | 311 | 0.07 | 660 | 1 | 1 | 0.5 | 0 | 0.5 | 0 | 0 | 0 | 0 | 0 |
| 2931 | 2 | 1 | 539 | 0.22 | 555 | 1 | 2 | 2 | 1 | 1.5 | 0 | 0 | 0 | 0 | 0 |
| 2953 | 2 | 1 | 418 | 0.22 | 418 | 1 | 0.5 | 1.5 | 2 | 2 | 0 | 0 | 0.5 | 0 | 0 |
| 2964 | 2 | 1 | 416 | 0.31 | 416 | 1 | 3.5 | 3.5 | 0.5 | 2 | 0 | 0 | 0 | 0 | 0 |
| 2965 | 2 | 1 | 416 | 0.23 | 416 | 1 | 0.5 | 2.5 | 2 | 1 | 0.5 | 0.5 | 0 | 0 | 0 |
| 2436 | 2 | 1 | 703 | 0.21 |  |  | 2.5 | 3 | 0 | 0.5 | 0 | 0 | 0 | 0 | 0 |
| 2437 | 2 | 1 | 703 | 0.07 |  |  | 1 | 1 | 0 | 0 | 0 | 0 | 0 | 0 | 0 |
| 2463 | 2 | 1 | 725 | 0.17 |  |  | 2 | 2 | 0 | 0.5 | 0 | 0.5 | 0 | 0 | 0 |
| 2464 | 2 | 1 | 725 | 0.15 |  |  | 1.5 | 1.5 | 0 | 1 | 0 | 0 | 0 | 0 | 1 |
| 2689 | 2 | 1 | 737 | 0.17 | 737 | 1 | 1 | 2 | 1.5 | 0.5 | 0 | 0 | 0 | 0 | 0 |
| 2694 | 2 | 1 | 736 | 0.21 | 737 | 1 | 1.5 | 2.5 | 1.5 | 0.5 | 0 | 0 | 0 | 0 | 0 |
| 2695 | 2 | 1 | 736 | 0.21 | 737 | 1 | 1 | 4 | 0 | 1 | 0 | 0 | 0 | 0 | 0 |
| 2696 | 2 | 1 | 707 | 0.11 | 764 | 0 | 1.5 | 1 | 0.5 | 0 | 0 | 0 | 0 | 0 | 0 |
| 2746 | 2 | 1 | 681 | 0.17 | 939 | 1 | 1.5 | 2.5 | 1 | 0 | 0 | 0 | 0 | 0 | 0 |
| 2798 | 2 | 1 | 785 | 0.14 | 928 | 1 | 1.5 | 1 | 1 | 0.5 | 0 | 0 | 0 | 0 | 0 |
| 2800 | 2 | 1 | 785 | 0.21 | 928 | 1 | 2.5 | 0.5 | 1 | 1.5 | 0.5 | 0 | 0 | 0 | 0 |
| 1968 | 2 | 1 | 1006 | 0.24 | 1281 | 1 | 2 | 3.5 | 0.5 | 1 | 0 | 0 | 0 | 1 | 0 |
| 1972 | 2 | 1 | 1031 | 0.5 | 1053 | 0 | 3.5 | 6.5 | 1 | 1 | 0.5 | 1 | 0 | 1 | 1 |
| 2182 | 2 | 1 | 728 | 0.26 | 734 | 1 | 2 | 2 | 1 | 2.5 | 0 | 0.5 | 0 | 0 | 0 |
| 2184 | 2 | 1 | 728 | 0.27 | 734 | 1 | 2 | 2.5 | 1.5 | 1 | 0.5 | 0.5 | 0 | 0 | 0 |
| 2743 | 2 | 1 | 681 | 0.28 | 939 | 1 | 1.5 | 3 | 1.5 | 1 | 0 | 0.5 | 0 | 1 | 1 |
| 2799 | 2 | 1 | 785 | 0.22 | 928 | 1 | 2.5 | 0.5 | 1 | 2 | 0.5 | 0 | 0 | 0 | 0 |
